# Skin Diseases and Syphilis

**Published:** 1896-09

**Authors:** 


### SELECTIONS AND ABSTRACTS.

### SKIN DISEASES AND SYPHILIS.

Edited by Dr. M. B. Hutchins.

### Vitiligo.

Dr. John V. Shoemaker, in a clinical lecture at the Medico-Chirurgical Hospital (*Am. Med. Surg. Bul.*, June 6, 1896), made some general remarks upon these causes of "turning white in spots." He says it may depend upon any disorder of the nervous system, and that the effects of malaria on the blood and nervous system often bring it on. He had seen a negro girl who was white on one side of the face and black on the other. He said that probably the most decided result in a large proportion of cases is produced by central and local galvanism. "The current will so stimulate the vaso-motor nerves and so impress the ganglionic center as to excite a more symmetrical distribution of pigment." He mentions the case of a mulatto woman who was cured by this means.

This disease is one of those which it is usually difficult or impossible to cure. If galvanism succeeds a *bête noire* of the dermatologist will disappear.

### The Superiority of Thiol to Ichthyol.

Dr. G. Morgan Muren (*N. Y. Med. Jour.*, June 27, 1896), expresses his preference for thiol. "Thiol is a definite synthetic compound." Ichthyol comes from a mineral deposit rich in sulphur. (Each gets its virtues, no doubt, from the sulphur elements which they both contain—the sulphur occurring in a convenient form.) Thiol has not the odor which is a drawback in the use of Ichthyol. He says it is non-irritating, and that it has a very soothing effect upon burns. The cases which he gives are not sufficiently numerous or decisive for the formation of a verdict, but if it can be demonstrated that Thiol is even as useful as Ichthyol the former will take the place, largely, of the latter, because it has no bad odor.

We used Thiol in the New York Skin and Cancer Hospital, but did not consider it as beneficial as Ichthyol.

---

### CHRYSAROBIN AND CHRYSOPHANIC ACID.

Dr. Walter G. Smith, in a paper read before the Dermatological Society of Great Britain and Ireland, May, 1896, went into the description of these two drugs and their action. It has been thought by many that the drugs were much alike in action and that the activity of Chrysarobin was due to its Chrysophanic Acid. (Many persons think one as good as the other.)

It is interesting to know, as these drugs constitute the best remedies for *Psoriasis* (locally applied), that actual experiments, side by side, demonstrate that Chrysarobin is far superior to Chrysophanic Acid in its action on the psoriatic lesions.

It is to be regretted that the results found by Dr. Smith were not reversed in view of his statement that good samples of goa powder, from which Chrysarobin comes, are now hard to obtain.—(From report in *British Journal of Dermatology*, July, 1896.)

---

### A CASE OF REINFECTION OF SYPHILIS.

Dr. Howard Paxton Collings, of Hot Springs, Ark., reports this case in *Journal of Cutaneous and Genito-Urinary Diseases*, August, 1896:

The patient gave a history of chancre and secondary symptoms eight years before. History correct as to the kind of symptoms. After thorough treatment, extending over two years, he had no symptoms for six years.

" Nearly nine years " after the first chancre, and twenty-eight days after exposure, the second chancre appeared on the penis, on the dorsal surface. Six weeks later "mucous patches" appeared about the anus. Moist "spots" appeared on the scalp after two weeks more. At the end of the tenth week a mucous patch beneath the tongue. Later, one on the tip of the tongue. Dark, excoriated, moist lesions appeared on the left calf and right gluteal region in three months. General adenopathy was observed when the doctor saw him at the end of the seventeenth week.

This is one of the best defined cases of reinfection which we have seen reported.

---

### CALCIUM CHLORIDE IN URTICARIA.

Dr. A. E. Wright reports two cases of urticaria treated by the administration of calcium chloride, having given as much as thirty grains at a dose. (*Brit. Journ. of Derm.*, March, 1896.) His theory is that these patients possess low blood coagulability, and that the drug increases it. If we can get a c. p. calcium chloride, and further experiments confirm Dr. Wright's results, we shall be the better able to control attacks of urticaria which are so troublesome in many people and so hard to manage.

---

### PRESCRIPTIONS.

*For Ringworm of the Skin:*

℞ Acid. pyrogallic ........................................ gr. xv.
Collodii ........................................ ℥ i.
M. Sig.—Paint on active spots to keep them covered.

*A Simple Dressing for Chancroid:*

℞ Calomel, hg. chl. mit.

OR,

Calomel, hg. chl. mit.
Bismuth, bism. subnit ........................................ aa.
M. Sig.—Keep the sore covered with this.

*A Salve for Late Syphilitic Sores:*

℞ Ung. hydrarg.,
Ung. zn. ox........................................ aa.
M. Sig.—Keep well on.

